# Lumbar spine intervertebral disc gene delivery of BMPs induces anterior spine fusion in lewis rats

**DOI:** 10.1038/s41598-022-21208-1

**Published:** 2022-10-07

**Authors:** Matthew E. Cunningham, Natalie H. Kelly, Bernard A. Rawlins, Oheneba Boachie-Adjei, Marjolein C. H. van der Meulen, Chisa Hidaka

**Affiliations:** 1 HSS Research Institute, Hospital for Special Surgery, 515 E 71st Street, New York, NY 10021 USA; 2 Weill Cornell Medical College, 1300 York Avenue, Lc501, New York, NY 10065 USA; 3 Meinig School of Biomedical Engineering and Sibley School of Mechanical and Aerospace Engineering, Cornell University, Ithaca, NY 14853 USA; 4 Hospital for Special Surgery, 535 East 70th Street, New York, NY 10021 USA

**Keywords:** Bone, Skeleton, Molecular medicine

## Abstract

Minimally invasive techniques and biological autograft alternatives such as the bone morphogenetic proteins (BMPs) can reduce morbidity associated with spinal fusions. This study was a proof-of-concept for gene-therapy-mediated anterior spine fusion that could be adapted to percutaneous technique for clinical use. Isogeneic bone marrow stromal cells genetically programmed to express b-galactosidase (*LACZ*, a marker gene), *BMP2*, *BMP7*, a mixture of *BMP2* and *BMP7* infected cells (homodimers, HM), or *BMP2/7* heterodimers (HT) were implanted into the discs between lumbar vertebrae 4 and 5 (L4/5) and L5/6 of male Lewis rats. Spine stiffening was monitored at 4, 8 and 12 weeks using noninvasive-induced angular displacement (NIAD) testing. At 12 weeks isolated spines were assessed for fusion and bone formation by palpation, biomechanical testing [four-point bending stiffness, moment to failure in extension, and in vitro angular displacement (IVAD)], faxitron x-rays, microCT, and histology. Progressive loss of NIAD occurred in only the HT group (*p* < 0.001), and biomechanical tests correlated with the NIAD results. Significant fusion occurred only in the HT group (94% of animals with one or both levels) as assessed by palpation (*p* < 0.001), which predicted HT bone production assessed by faxitron (*p* ≤ 0.001) or microCT (*p* < 0.023). Intervertebral bridging bone was consistently observed only in HT-treated specimens. Induced bone was located anterior and lateral to the disc space, with no bone formation noted within the disc. Percutaneous anterior spine fusions may be possible clinically, but induction of bone inside the disc space remains a challenge.

## Introduction

Spinal fusion is the definitive method of treatment for progressive spine deformity, instability, and in certain presentations of spinal infections or tumors. However, spine fusion is associated with numerous risks and intraoperative complications including blood loss, infection, or neurological injury^1–4^ and postoperative complications including pseudarthrosis^5, 6^, instrumentation failure^7, 8^, or junctional degeneration^9, 10^. Efforts to minimize morbidity associated with spinal fusions have involved use of minimally invasive techniques^11, 12^, modern osteoinductive molecules including the bone morphogenetic proteins (BMPs)^13, 14^, and recombinant parathyroid hormone analogs^15–18^.

Animal models for spine fusion have been instrumental in the development of these advances, particularly as regards osteobiological agents such as the BMPs. Basic and translational comparative research established the BMPs as being at least as effective as iliac crest autograft bone (ICBG) in specific situations^19, 20^, and have led to the use of BMPs for spine fusion clinically to minimize ICBG donor site pain, pseudarthrosis^14, 21^, and complications in general^22^. Use of BMP through an anterior approach in a metal cage for 1 level lumbar fusions, as approved by the FDA in 2002, provided patients with very predictable results, and lead to its wide adoption for on^23–25^ and off-label^26–28^ use. BMPs became popular in minimally invasive applications, further speeding patient recovery and increasing tolerance of fusion surgery due to decreasing morbidity associated with the procedures^29, 30^, despite instances of ectopic bone formation^31, 32^, and postoperative radiculopathy following direct BMP contact with nerve roots^33, 34^. Safe and effective percutaneous BMP injections for spinal fusion holds promise as a technique to minimize morbidity. In comparative models this concept has been shown to be feasible for posterior spine fusion using gene-therapy BMP delivery^35–39^. However, percutaneous approaches have resulted in spine fusions at adjacent levels, most likely reflecting either dispersion of the BMP injectate or exuberant heterotopic ossification (HO) from a contained injection site. The intervertebral disc space (IVD) is a potentially better confinement option, because BMP delivery and bone induction within the nucleus pulposus (NP) of the IVD would allow the annulus fibrosus (AF) to act as a localization barrier^40, 41^.

Potential issues with the small-animal IVD as a target for delivery include its relatively small size^42^ and potential for treatment leakage^43, 44^, and specifically for fusion studies, the NP has been suggested to be anti-osteogenic^45–47^. Prior lumbar IVD implantation to generate anterior fusion included open approaches and chemonucleolysis^48, 49^, active/mechanical NP removal^50^, or extensive AF trauma to allow passive NP egress^51^ in combination with BMP delivery, and the induced bone formed predominantly anterior to the implanted disc space, including in the anterior longitudinal ligament and superficial anterior AF. As regards delivery of BMPs through an open approach to an otherwise intact IVD (NP not intentionally removed), only one group has reported bone formation with BMP7 delivery, despite their intention to drive canine IVD regeneration^52^. All other studies reviewed that delivered factors or gene-therapy through open approaches to intact IVDs to date have shown the NP cells to be reactive to BMPs in such a way to drive glycosaminoglycan and proteoglycan production, restore IVD height and MRI hydration signal in damaged IVDs, and to not induce bone production within the IVD^53–58^. Most of the BMP delivery to IVDs has been of homodimeric forms of BMP2, BMP7 and BMP-14, but more recent reports using the hyper-osteoinductive^59–61^ heterodimer BMPs, have also demonstrated disc regenerative and protective effects and not fusions or bone induction *in vitro*^58^ and *in vivo*^62^, or absent in vivo disc/bone anabolic effects^63^. BMP heterodimers strongly increase fusion in animal posterolateral fusion models^59, 64, 65^, but their effectiveness has not been reported for comparative anterior IVD fusions.

In the present study we hypothesized: (1) intra-discal delivery of cells that are genetically modified to express BMPs would drive bone formation within or proximate to intervertebral discs prepared by endplate perforation, (2) BMP-induced bone would lead to spinal fusions, and (3) the relative strength of osteoinductive signals from BMP-2, -7, or the -2/7 heterodimer delivered would demonstrate differential effects on bone formation and fusion.

## Materials and methods

Animal surgeries and primary cell cultures were performed under an HSS IACUC-approved protocol, and following ARRIVE guidelines. All experiments were performed under HSS  Comparative Lab Animals Services and HSS Research Division guidelines and regulations. Lewis rat (*n* = 15 male donors) bone marrow mesenchymal stromal cells (BMSCs)^43, 66^, and adenoviral (Ad) vectors for b-galactosidase (*LACZ*), human *BMP-2* and *hBMP-7* were generated^59, 66, 67^. BMSCs were transduced with 10^5^ particle units (pu)/cell of Ad 18–24 h prior to surgery, with gene expression verified by representative aliquots 3 days later: X-gal staining for LACZ^68^, or ELISA for BMP-2 (R&D Systems Inc., Minneapolis, MN) and BMP-7 (Alpha Diagnostic International, San Antonio, TX)^59, 69^. BMP2/7 heterodimer was measured by immobilization on anti-BMP2 conjugated wells (BMP2 ELISA kit), and detection by the anti-BMP7 secondary (BMP7 ELISA kit). Animals implanted with cells lacking transgene expression were euthanized. Programmed cells were implanted into the L4-L6 disc spaces of 105 male Lewis rats through an open ventral transperitoneal approach, as previously described^43, 70^. Endplate preparation (2–3 punctures/disc) and cell implantations were done using only hypodermic needles (interventions that could be done in humans percutaneously), with BMSCs delivered at the previously optimized dose of 10^6^ cells in 25µL^43^. A power calculation with alpha set at 0.05, beta at 0.8, and an average treatment effect of 50% (estimated using our prior posterior heterodimer fusion results)^59^ revealed that 12 animals would be required for each group. At least 15 rats per group were injected with BMP-2 (Ad-*hBMP2* infected cells expressing BMP2 homodimers), BMP7, “homodimers” (HM – equally mixed BMP2 and BMP7 cells after trypsinization), and “heterodimers” (HT – cells doubly infected with 10^5^ pu/cell of each Ad-*hBMP2* and Ad-*hBMP7*) expressing cells. Rats in the control group were injected with LACZ (Ad-*LACZ* infected) cells. Animals were recovered, and allowed ad libitum food, water and activity.

Spine stiffening over time and fusion endpoints were measured. Passive segmental lumbar motion was monitored using noninvasive in vivo angular displacement (NIAD)^71^ at weeks 4, 8 and 12 (Table 1). The effect of time on NIAD was assessed after data stratification by treatment. At 12 weeks, euthanized animals were imaged in AP and lateral projections to evaluate bone formation, probability of fusion, and to assess distant heterotopic ossification (HO). Graded segmental spine *bone formation* and *fusion likelihood* were rated independently for L4/5 and L5/6 on lateral radiographs using a method adapted from Petersen et al.^72^ (Table 1 and Supplementary Fig. S1). Graded bone formation was tested by comparing rater’s sums (0–6, nominal data) using non-parametric statistics. Categorical radiographic fusion was graded as bridging bone across L4/5 and/or L5/6 on lateral radiographic images (Table 1, Supplementary Fig. S1)^59, 66^. Images were also used to assess the lungs and liver^44^ and remainder of the abdomen^73^ for HO, but none was noted. Spines were explanted *en bloc* L3-S1 and stripped of soft tissues apart from the surgical zone, and were assessed by manual palpation, the “gold standard” for experimental rat spine fusion assessment^72, 74–76^. Separate scores at L4/5 and L5/6 were rated as “fused” = no motion, or “not fused” = motion detected (Table 1)^59, 77^.

**Table 1:** NIAD, Manual Palpation and Radiographic Evaluation Rubric.

	NIAD	Palpation for fusion	Graded radiographic fusion	Categorical radiographic fusion
Observers	3, blinded	3, blinded	3, blinded	3, blinded
Grading	Twice, 2 weeks apart	Once*	Once**	Once*
Interobserver agreement	Yes, ICC	Yes, Fleiss K	Yes, Fleiss K	Yes, Fleiss K
Intraobserver agreement	Yes, ICC	No	No	No
Data	Continuous, non-normal	Dichotamous	Dichotamous	Dichotamous
Data testing	Kruskal-wallace	Fisher exact	Fisher exact	Fisher exact

*Raters grade as fused or not, and specimen is assigned status by rater majority.

** Raters grade 0–2, scores are summed, and sum determines status. Sum ≥ 5 is fused.

Ten spines from each group underwent in vitro angular displacement (IVAD), and four-point bending non-destructive mechanical stiffness and failure testing^71^. Four-point bending was performed over the combined L4-L6 segment with loads applied at 0.5 N/sec to a maximum load 4 N (ELF 3200, EnduraTec, Eden Prairie, MN), for 5 cycles with stiffness measured on the 5^th^ cycle (Supplementary Fig. S2). Number-coded specimens from all groups were pooled and processed in random order, and each specimen was tested in random sequence in each of the 4 directions: right and left lateral bending, extension and flexion. IVAD was measured from a digital photograph (Nikon D70, Melville, NY, USA) taken at maximal deflection in each of the 4 directions during the 5th cycle (Supplementary Fig. S2)^71^. Composite coronal IVAD was the sum of a specimen’s right and left IVAD measures, and composite sagittal IVAD was the sum of flexion and extension IVAD measures. Failure bending moment in extension and failure location (L4/L5 or L5/L6 disc) were recorded in the destructive biomechanical tests. Bending failure locations were compared using the one-sample T-test for proportion (https://www.medcalc.org/calc/test_one_proportion.php).

Five spines from LACZ-, HM- and HT-treated animals were used for micro-CT and histology assessment of bone formation and quality. Samples were fixed, washed with PBS, and quantitative microCT scans of rat spines from L3-S1 were taken at 17 µm isotropic resolution (MS-8, GE Healthcare, London, Ontario, Canada), as previously described^59, 77, 78^. Total volume of induced bone was measured, as was cancellous architecture and mineralization parameters of the fusion mass or non-fusion mass cancellous bone within the vertebral bodies (Supplementary Fig. S1). Following microCT, samples were decalcified in EDTA, paraffin embedded, sagittally sectioned at 5 µm, stained, and assessed under light microscopy (Nikon Eclipse E800 microscope, Nikon Instruments Inc., Melville, NY) for endpoints (Table 2).

**Table 2:** Histology Assessment Rubric.

Criteria	Section stain	Description
Marrow quality	H&E	Hematopoietic (red) – normal appearing, -or- Fatty – frequently found with BMP treatment^118–120^
Bone Spurs or Bridging Bone	H&E	Anterior bone formed adjacent to the growth plate beneath the anterior longitudinal ligament, directed towards the disc space that: does not bridge the disc (bone spur), or does (bridging bone, or fusion mass)
NP cell cloning	H&E and Alcian blue	Multiple NP cells in a single lacuna
Cartilage and proteoglycans	Alcian blue	NP, AF and endplate (EP) staining intensity (deep blue vs lighter or no blue) and pattern (biphasic vs monophasic)
Endplate damage and organization	Alcian blue	EP puncture site (+ / −) and relative organization
Notochordal cell presence	Alcian blue	Physaliphorouscells (eosinophillic appearing NP cells in Alcian Blue stained sections)^121^
Disc herniations	All stains	Presence/absence of disc herniations (Schmorl’s Nodes) through the EPs, with disc material in the vertebral bodies

Data not meeting the normality assumptions of parametric tests (NIAD, IVAD, four-point biomechanics), ordinal data (Graded bone induction score), and data with small number of samples and concern for not meeting assumptions of parametric testing (micro-CT) were compared using the non-parametric Independent Samples Kruskal-Wallace test, with Dunn-Bonferroni post-hoc pairwise testing (SPSS v.22, SPSS Inc., Chicago, IL). Dichotomous data (fusion status, microscopy findings) was tested using the Fisher Exact test with post-hoc pairwise testing and significance adjusted by Bonferroni correction (https://www.socscistatistics.com/tests/fisher/default2.aspx). ICC was single measures 2-way mixed-effect test for consistency^71^ (SPSS), and Fleiss’ Kappa was calculated (https://www.statology.org/fleiss-kappa-excel). Data correlation was assessed by Pearson’s r (continuous data) and with Spearman’s r (non-continuous) (SPSS). Box plots represent median (belt), 75^th^ and 25^th^ percentiles (top and bottom of box), and maximum and minimum data values (whiskers).

## Results

### BMP2/7 heterodimer treatment decreased NIAD over the 12 week time course

**Figure 1:**
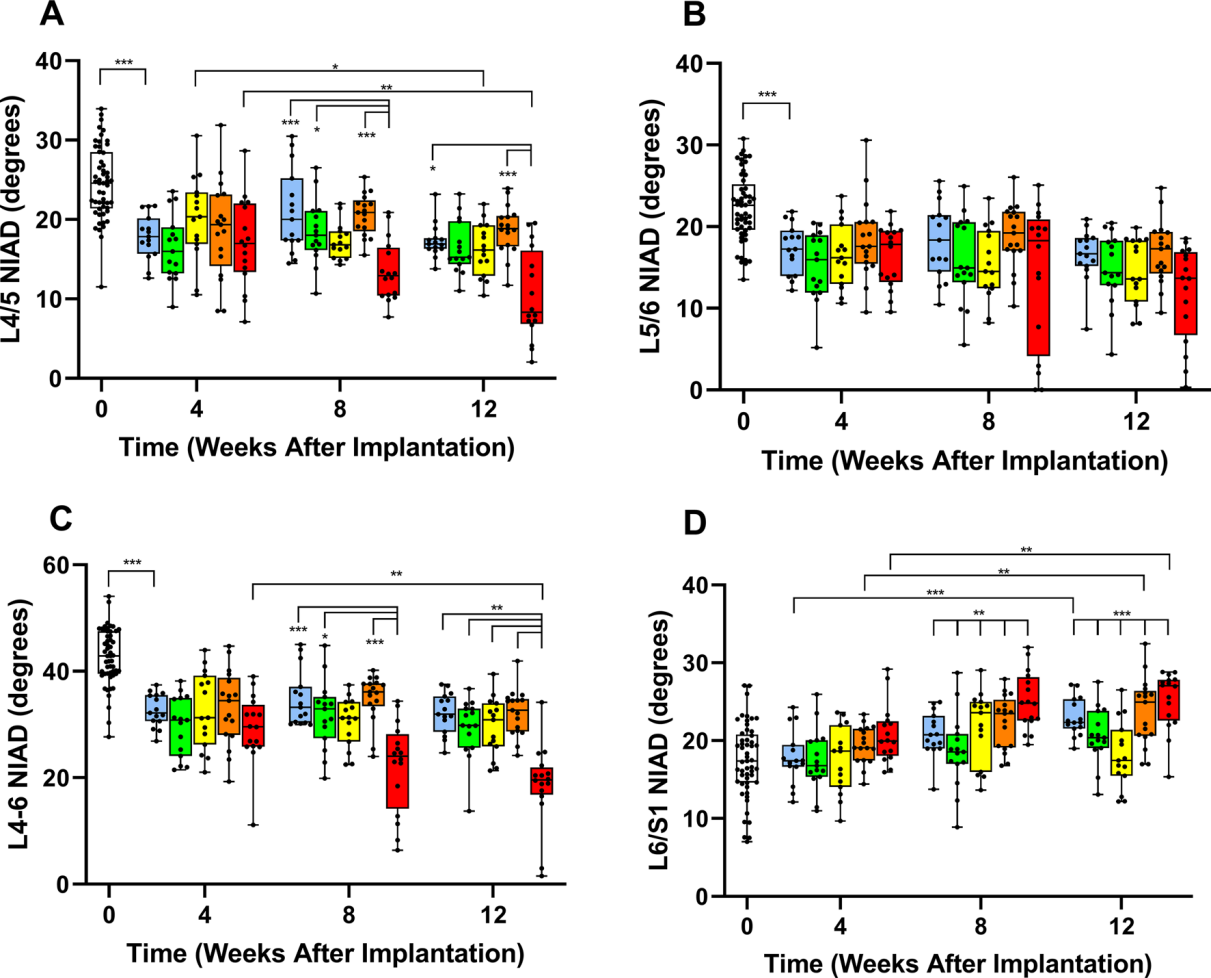
Coronal noninvasive in vivo angular displacement (NIAD) of rat lumbar motion segments had the greatest decline in mobility for the BMP2/7 heterodimer (HT)-treated spines at (**A**) lumbar 4/5 (L4/5), (**B**) L5/6, and (**C**) combined L4-6 segment at 4, 8 and 12 weeks following intradiscal administration of genetically-programmed bone marrow stromal cells. (**D**) Increased mobility was detected in all groups at the L6/S1 segment over the same time course, likely due to stiffening at the L4-6 levels. Time of assessment (horizontal axis) and treatment (white square is Naïve, blue square is LACZ, green square is BMP2, yellow square is BMP7, orange square is mixed BMP2 and BMP7 [homodimers, HM], red square is BMP2/7 [heterodimers, HT]) are indicated. Individual data points are shown, box plots represent treatment-group median, 25th and 75th percentiles and whiskers denote minimum and maximum data points. Significance: *** *p* ≤ 0.001, **p ≤ 0.01, and *p ≤ 0.05.

At 4 weeks post-procedure NIAD decreased 25% in the LACZ (negative control) group compared to historical non-operative controls for the L4/L5, L5/L6 and L4-L6 segments, but not the L6/S1 segment (Fig. 1). By 8 weeks the HT-treated group had significantly less residual NIAD (at L4/5 and L4-6) than LACZ, BMP2 and HM, but was not different from BMP7 (Fig. 1A and C). At 12 weeks for L4/5, HT NIAD was less than LACZ and HM but not different from BMP2 or BMP7 (Fig. 1A). At 12 weeks for L4-6, HT NIAD was lower than all other groups (Fig. 1C). There were no treatment effects for L5/6 at 8 weeks or 12 weeks (Fig. 1B). The NIAD increased at L6/S1 at 8 weeks and 12 weeks, as anticipated with stiffening/fusion of the L4-L6 lumbar segment (Fig. 1D), but post-hoc testing revealed no intergroup treatment effects. Decreased L4/5 NIAD was time dependent only for BMP7 and HT treatments (Fig. 1A). For the combined L4-6 segment NIAD decrease was time-dependent for only the HT treatment group (Fig. 1C). At L5/6 NIAD had no significant time dependence by treatment group (Fig. 1B). At L6/S1 increased NIAD was time dependent for LACZ, HM and HT, but not BMP7 or BMP2 (Fig. 1D). ICC for 3 rater inter-rater reliability was *good* at 0.881 (95%CI 0.830–0.919, *p* < 0.001), and ICCs for each rater’s test–retest reliability was *good* to *excellent* (Table 3).

**Table 3:** Measurements of Rater Agreement for Post-operative Sample Assessments.

Inter-rater	ICC	95%CI	*p*-value	Intra-rater	ICC	95%CI	*p*-value
Continuous data—assessed by ICC*							
R1 vs. R2	0.829	0.810–0.846	< 0.001	R1 vs. R1	0.901	0.857–0.932	< 0.001
R2 vs. R3	0.885	0.822–0.926	< 0.001	R2 vs. R2	0.905	0.833–0.947	< 0.001
R3 vs. R1	0.846	0.764–0.901	< 0.001	R3 vs. R3	0.863	0.790–0.912	< 0.001

3-Rater test	Fleiss’ K	3-Rater Test	Fleiss’ K	3-Rater Test	Fleiss’ K		
**Ordinal and dichotamous data—assessed by Fleiss’ Kappa**							
Fusion by palpation	0.811	Categorical radiographic fusion	0.844	Graded radiographic fusion	0.762		

* Interpreted as *poor* (< 0.5), *moderate* (0.5 to 0.75), *good* (0.75 to 0.9), and *excellent* (> 0.9).^122^.

** Interpreted as *poor to fair* (< 0.4), *moderate* (0.41 to 0.6), *substantial* (0.61 to 0.8) and *almost perfect* (0.81 to 1.0)^123^.

### IVAD and mechanical testing in four-point bending independently confirms and correlates with NIAD BMP HT-treatment increased spinal stiffness

**Figure 2:**
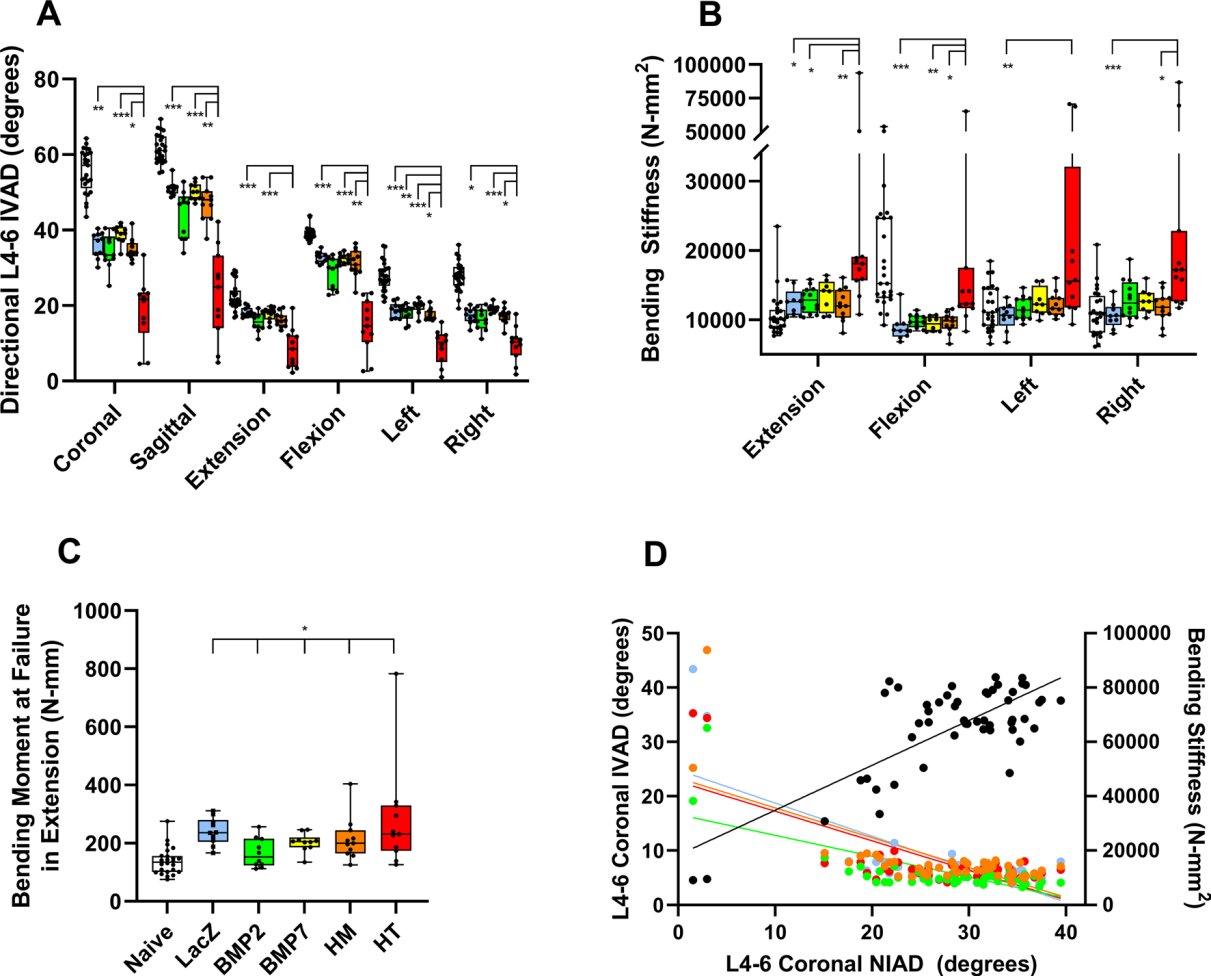
In vitro angular displacement (IVAD) (**A**) and four-point bending (**B**) assessments showed greatest stiffness induction in BMP2/7 heterodimer (HT) treated spines. Spines were assessed for IVAD and four-point bending over the combined lumbar segment 4–6 (L4-6) at 12 weeks after intradiscal delivery of genetically programmed bone marrow stromal cells. The bending moment at failure in extension (**C**) showed a treatment effect, but post-hoc testing did not reveal a unique treatment. In (**A**)–(**C**) treatments correspond to color (white square is Naïve, blue square is LACZ, green square is BMP2, yellow square is BMP7, orange square is mixed BMP2 and BMP7 [homodimers, HM], red square is BMP2/7 [heterodimers, HT]). Individual data points are shown, box plots represent treatment-group median, 25th and 75th percentiles and whiskers denote minimum and maximum data points. Significance: *p* ≤ 0.001 (***), *p* ≤ 0.01 (**), and *p* ≤ 0.05 (*). (**D**) scatter plot of paired IVAD:NIAD (black circle) and NIAD:four-point bending stiffness (orange circle is extension, green circle is flexion, red circle is right bend, and light blue circle is left bend). Note that the infrequently observed double-level fusions represent the low NIAD value specimens on the left side of the graph.

At 12 weeks the L4-6 segment of the HT-treated animals was less mobile than all other groups except BMP2 (Fig. 2A) for composite coronal and sagittal IVAD. Correlation of in vivo (NIAD) and coronal in vitro (IVAD) measurements over the combined L4-6 segment was significant, with Pearson’s coefficient of r = 0.751 (*p* < 0.001) (Fig. 2D). Non-destructive four-point bending stiffness testing had inter-group treatment effects in each direction with only HT samples having inter-group differences (Fig. 2B). Load to failure in extension was affected by treatment, but post-hoc testing did not identify a uniquely different treatment (Fig. 2C). Failure location was dictated by the fusion status of the samples in the HT group, and all non-fused samples had an increased frequency of failure at the L5/6 level (32 out of 42, *p* < 0.001 with 95%CI [60.55, 87.95]). Significant correlation (*p* < 0.001 for each) was noted in 4 directions between IVAD and four-point stiffness, with Pearson’s r =  − 0.607 in extension, r =  − 0.672 in flexion, r = -0.712 in left bending, and r = -0.655 in right bending. Similarly, L4-6 (coronal) NIAD correlated (*p* < 0.001 for each) with four-point biomechanics in extension (r =  − 0.692), flexion (r =  − 0.690), left bending (r =  − 0.735) and right bending (r =  − 0.727), whereas correlation with failure moment in extension was significant (*p* = 0.005) but weaker (r =  − 0.385) (Fig. 2D).

### BMP HT treatment resulted in an increased frequency of spinal fusion assessed at 12 weeks by manual palpation and radiography

Fusion assessed by palpation at L4/5 occurred in 69% of HT spines, and at one or both levels of HT spines 94% of the time (15/16). At L4/5 HT treatment resulted in fusion more frequently than in any other treatment (*p* = 0.006), and no other comparative intergroup treatment effects were observed (*p* = 1). Palpation at L5/6 also revealed that HT (7/16 or 31%) outperformed all other groups (1/62 or 2%) (*p* < 0.001), but post-hoc pairwise testing did not identify intergroup differences after Bonferroni correction (*p* = 0.37) (Table 4). Palpation-assessed fusion for the entire population was not different at L4/5 (12/78) than at L5/6 (8/78) (*p* = 0.47), and palpation-assessed fusion success was not different by level for the HT-treated group (*p* = 0.28). Categorical radiographic fusion also occurred most frequently in HT as compared to all other groups at L4/5 (69% vs. 0–6.7%, *p* = 0.006) with no other inter-group treatment effects (*p* = 1), but L5/6 did not show a treatment effect difference (18.8% vs. 0%, *p* = 0.599) (Table 4). Similarly, graded radiographic fusion for L4/5 occurred most frequently in the HT group at L4/5 than all other groups (*p* ≤ 0.001) with no other intergroup effects (*p* = 1) (Table 4), but no treatment effect was observed for L5/6 (*p* = 0.101). Fleiss’ kappa for inter-rater agreement was *almost perfect* for palpation and categorical radiographic fusion, and was *substantial* for graded radiographic fusion (Table 3). Spearman’s rank-order test showed strong correlation between palpation and categorical radiographic fusion (r = 0.739, *p* < 0.001), and palpation versus graded radiographic fusion (r = 0.665, *p* < 0.001).

**Table 4:** Multimodality Fusion Assessments at Lumbar Levels 4/5 (L4/5) and 5/6 (L5/6) 12 Weeks Following Intradiscal Injection of Genetically Programmed Bone Marrow Stromal Cells.

Treatment group	Number in group	Manual Palpation**		Categorical XRay**		Graded XRay**	
		L4/5	L5/6	L4/5	L5/6	L4/5	L5/6
LACZ (marker)	15	0	0	0	0	0	0
BMP2	15	1	1	1	0	0	0
BMP7	15	0	0	0	0	0	0
BMP2 and 7 Homodimers	17	0	0	0	0	0	0
BMP2/7 Heterodimer	16	11	7	11	3	13	4

*Samples were considered fused if in vivo angular displacement (NIAD) measurement was 3 standard deviations below pre-operative average value. ** Palpation and radiographic fusion was determined as described in Materials and Methods.

### Decreased mobility and increased frequency of fusion was the result of bone formation around the L4/5 and L5/6 IVDs

Bone formation was appraised by faxitron, microCT and histology. The greatest amount of bone formed in the HT-treated spines, with each method showing fusion bone primarily anterior and laterally to, but not within the disc space. Assessment by faxitron demonstrated abundant bone in only the HT group, with moderate amounts of bone also observed in spines from BMP2 and HM groups (Table 5). Significantly more spinal levels had moderate or abundant bone production in HT than any other group (*p* ≤ 0.001), and no other inter-group comparisons showed differences for bone formation (*p* ≥ 0.445). Quantification by microCT for the L4-6 segment showed the greatest bone volume in the HT group (average 35.2 ± 15.5 mm^3^), and less in the LACZ (7.6 ± 1.3 mm^3^) and HM (9.6 ± 2.6 mm^3^) groups, demonstrating a treatment effect (*p* = 0.023). Post-hoc testing confirmed a difference between HT and LACZ (*p* = 0.032) but not HT and HM (*p* = 0.093), and none between LACZ and HM (*p* = 1). Nearly identical treatment effects for microCT were obtained by level at L4/5 (p = 0.023) and L5/6 (*p* = 0.018) with post-hoc testing only showing HT greater than LACZ (*p* = 0.032 and *p* = 0.018, respectively) and no significance for the other 2 comparisons. Spearman’s correlation showed strong agreement between faxitron and microCT assessment of bone quantifications for combined L4/5 and L5/6 data sets with r = 0.722 and *p* ≤ 0.001.

**Table 5:** Faxitron-assessed Bone Formation at Lumbar levels 4/5 (L4/5) and 5/6 (L5/6) at 12 Weeks Following Intradiscal Injection of Genetically Programmed Bone Marrow Stromal Cells.

Treatment group	Number in group	Minimal		Moderate		Abundant	
		L4/5	L5/6	L4/5	L5/6	L4/5	L5/6
LACZ marker	15	15	15	0	0	0	0
BMP2	15	11	13	4	2	0	0
BMP7	15	15	15	0	0	0	0
BMP2 and 7 Homodimers	17	16	16	1	1	0	0
BMP2/7 Heterodimer	16	3	5	0	7	13	4

Bone formation was graded 0–2 by 3 observers as described in Materials and Methods, and summed score of 0–2 = Minimal, 3–4 = Moderate, and 5–6 = Abundant bone production.

Bone quality assessment at the fusion site by microCT showed higher bone mineral content (BMC) and tissue mineral content (TMC) at L5/6 in HT than any other group (*p* = 0.026). However, no treatment effect was observed for bone mineral density (BMD), tissue mineral density (TMD) or bone volume fraction (BVF). At L4/5 we detected no treatment effect for BMC, BMD, TMC, TMD or BVF. The cancellous bone parameters (BMC, BMD, TMC, TMD, BVF, and bone architecture measures—trabecular thickness, trabecular number) were not different for L3, L4, L5, or S1 vertebrae by treatment group.

**Figure 3:**
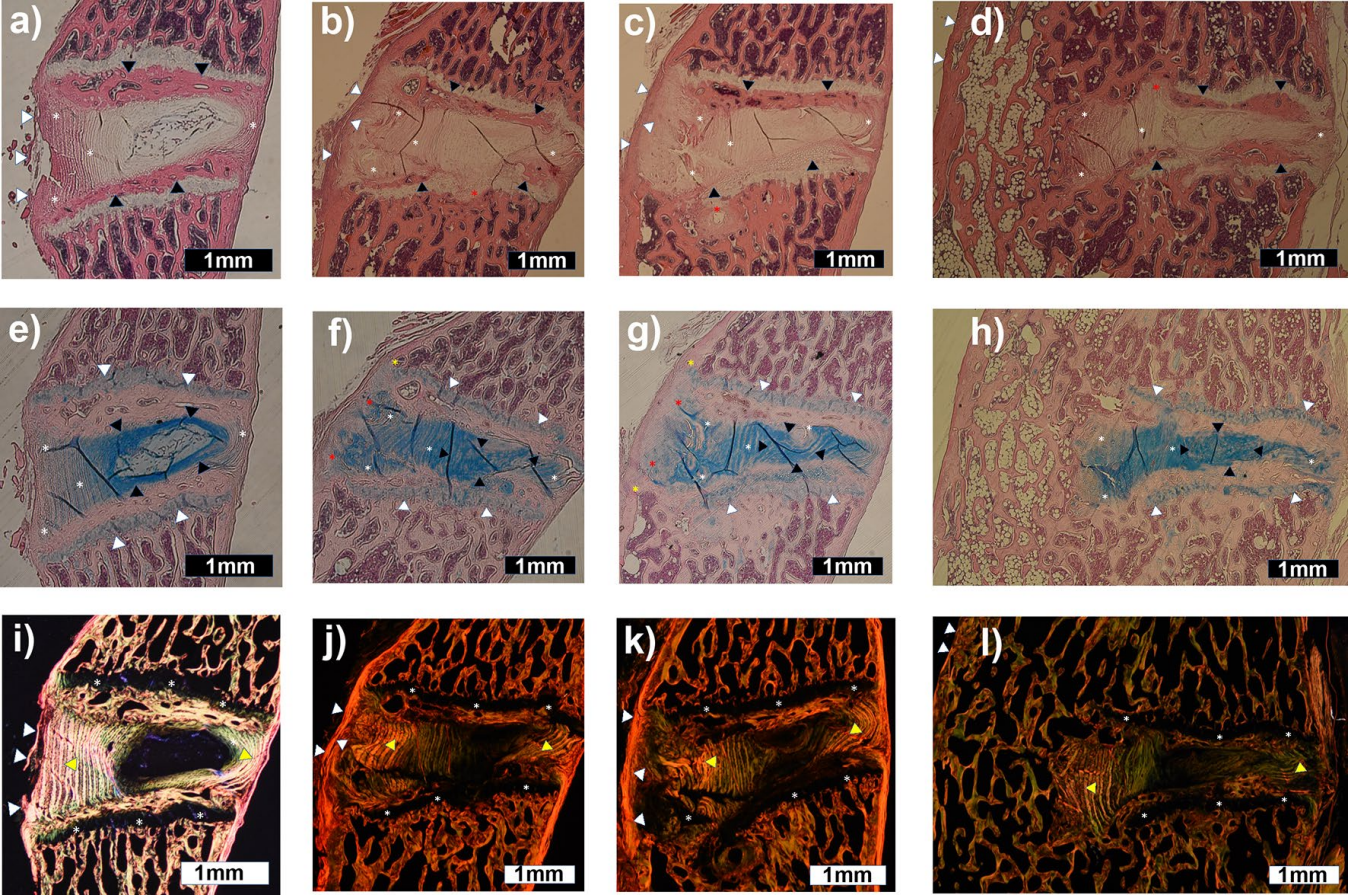
Microscopic morphology of fusion bone and intervertebral disc space tissues 12 weeks after intradiscal implantation of genetically programmed bone marrow stromal cells. Spines were explanted, decalcified, paraffin embedded, sagittal sectioned, and assessed for: general architecture (H&E stain—**A**, **B**, **C** and **D**), proteoglycan/cartilage (Alcian Blue stain—**E**, **F**, **G** and **H**), or collagen (Picrosirus Red stain and circular polarized light—**I**, **J**, **K** and **L**). Representative images from specimen 29 from a non-operated normal L3/4 level (**A**, **E** and **I**), specimen 26 LACZ (marker gene)-treated L4/5 level (**B**, **F** and **J**), specimen 29 BMP2 and BMP7 homodimer (HD)-treated L4/5 level (**C**, **G** and **K**), and specimen 24 BMP2/7 heterodimer (HT)-treated L5/6 level (**D**, **H** and **J**) are shown. H&E stained images are marked to show: anterior longitudinal ligament (ALL) (white triangle) (partially discontinuous distally as an artifact of preparation in A), annulus fibrosus (AF) (white asterisks), and epiphyseal bony endplates (black triangle). Alcian Blue stained images are marked to show: endplate growth plates (white triangle), anterior and posterior AF lamellae (white asterisks), and nucleus pulposus (NP) (black triangle). Picrosirus Red polarized images are marked to show: bright yellow/red and green coloring of high collagen content structures (ALL [as white triangle], AF [as yellow triangle], and vertebral body bone network) and black coloring of low collagen content (growth plates [as white asterisks], NP space, and bone marrow compartments in the vertebral bodies). (**A**)–(**H**) shows annulus fibrosus (AF) and nucleus pulposus (NP) are well stained and generally preserved. (**A**) and (**E**) shows the non-operated NP has a central volume that stains lighter with Alcian Blue and contains small eosinophilic (physaliphorous) cells, which are not seen in the more homogeneously dark blue staining NP compartments of the operated levels (**B**–**D** and **F**–**H**). Note scar formation and disorganization of the bony and cartilaginous growth plate epiphyses (red asterisks in **B**, **C** and **D**) at what were interpreted to be endplate puncture sites. Comparing (**E**) with (**F**) and (**G**) shows additional operative changes including: new cartilage-appearing cells between the ALL and anterior fibers of the AF (red asterisks) staining similarly and in apparent continuity with the cartilaginous growth plate, and new vertebral body bone extending anterior to the anterior AF that is minor in Panel F but more noticeable in G (yellow asterisks). D & H shows impressive bone production anterior to the disc space in an HT treated specimen, contiguous with the marrow space, and fatty appearing marrow. Segments are shown with top left of sections being ventral and proximal, magnification at 20 × , and scale bar representing 1 mm.

**Figure 4:**
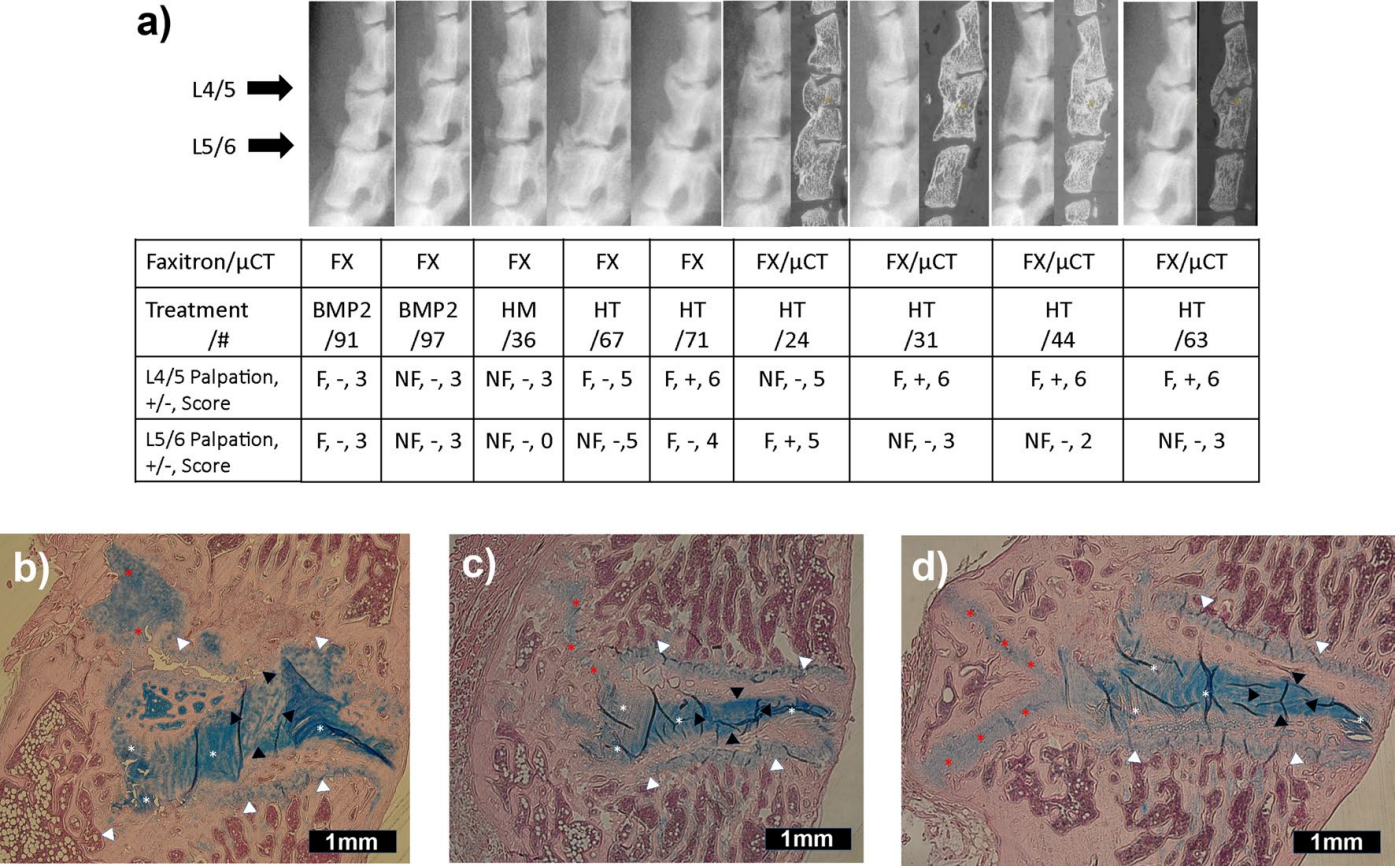
Fusion status assigned by palpation, radiography (faxitron or micro-CT) and histology don’t always directly translate at 12 weeks after disc-implantation of BMP-expressing cells. (**A**) Lateral faxitron and midsagittal micro-CT images (when available) for several specimens where palpation (“Palpation” – fused (F) / not fused (NF)) was inconsistent or non-correlative to dichotomous radiographic [fused (+)/not fused (−)] or the 0–6 sum score (sum shown, 0–4 is not fused and 5–6 is fused). BMP treatment and specimen number are indicated to allow cross-comparison with Figs. 3, 4B–D, and Supplementary Fig. S1. Note that segmental kyphosis or disc height loss of an L4/5 or L5/6 segment tends to correlate with anterior fusion mass status (compare levels fused/not for specimens 24, 31, 44, and 63). (**B**)–(**D**) Alcian blue stained histology sections demonstrating cartilage clefts through the anterior induced bone fusion masses, noted in some of the other heterodimer-treated specimens not shown in Fig. 3. Images are marked to show: endplate growth plates (white triangle), anterior and posterior AF lamellae (white asterisks), nucleus pulposus (NP) (black triangle), and presence of cartilage clefts (red asterisks). Images are of the L4/5 level of each specimen, (**B**) shows specimen 24, (**C**) shows specimen 31, and (**D**) shows specimen 63. All images are oriented with top left being ventral and proximal, magnification is 20 × , and scale bar represents 1 mm.

Light microscopy revealed bridging bone between vertebral bodies in 75% (6/8) of specimens from the HT treated spines, but in none of any of the other treatments (*p* = 0.021) (Fig. 3, Table 2). Comparing non-operated L3/4 or L6/S1 levels to the operated levels showed anterior endplate osteophytes extending past the anterior fibers of the AF in 14/24 specimen-levels (58%), with 1/8 (12.5%) in LACZ, 5/8 (62.5%) in HM and 8/8 (100%) for HT (*p* = 0.0042 for LACZ versus HT, other pairwise comparisons were not significant). The medullary space of the HT fusion bone was in continuity with the intramedullary canal of the vertebral bodies, suggestive of extensive fusion callous remodeling, and the marrow space of the induced fusion bone was noted to have extensive fatty infiltration (Fig. 3D, H). No obvious bone formation was observed within the AF lamellae of the fused levels. Bone was not present in the IVD interior nor was there consistent evidence of vascular or marrow elements within the disc space or in apposition to the endplate needle puncture sites. Alcian blue staining of the NP was preserved for all treatments (Fig. 3E–H), but suggestion of disc height loss and kyphosis across the disc space was noted in HT-treated fused levels only (Fig. 3 and 4A), presumed due to fusion tethering anteriorly and continued growth through the epiphyses. Alcian Blue ( +) cartilage-appearing cells were noted between the anterior longitudinal ligament (ALL) and anterior AF lamellae, were contiguous with the juxtaposed cartilaginous growth plates, and were observed in LACZ, HM and HT treatment groups (Fig. 3F–H); cartilage clefts were noted in some of the HT bridging fusion specimens (Fig. 4), also contiguous with the cartilaginous growth plates, and were thought to represent the cells described between the ALL and anterior AF noted in the other treatment groups. Disorganization of the fibers of the annulus fibrosis and cartilage endplates was noted in the surgical specimens only, but was not specific to treatment conditions, as were also the losses of NP biphasic Alcian blue staining and physaliphorous cells, and the general increase in NP cell number and examples of NP cell cloning.

## Discussion

This study describes a rat model that is a proof of concept for percutaneous anterior spine fusions in humans. To our knowledge, this report is the first reproducible anterior spine fusion in a comparative animal model without extensive damage or partial mechanical removal of the IVD. Intradiscal injection of bone marrow-derived stromal cells genetically modified to express BMP2/7 HT resulted in increasing spinal stiffness with successful spinal fusion of one or both levels in 94% (15/16) of treated animals and abundant bone formation anterior and lateral to the IVD. Differential osteoinduction was demonstrated, with little to no bone formation or spine fusion with the LACZ negative control, and some bone formation but no consistent fusion with delivery of the BMP2 and BMP7 homodimers, alone or in combination, and the most bone and fusion noted with HT treatment. Fusion bone was remodeled, with marrow cavity continuous with that of the host vertebral bodies. Notably, despite our concept to use the AF as an anatomical containment for the IVD treatment delivery and bone induction, no bone was present in the IVD space.

Our finding that fusion and bone formation occurred consistently only in the BMP2/7 HT group is in agreement with previous findings. BMP heterodimers are known to be more potent osteoinductive factors in certain settings^59, 60, 64, 65^, even when expressed at lower levels than their homodimer counterparts^64^. This super-osteoinductive capacity of the heterodimers may be due to their ability to escape sequestration by noggin^69^, their inducing less inflammation than by homodimer BMPs^79^, their altered use of specific cell surface receptors^80, 81^, or by their driving different intracellular signaling pathways^82, 83^. Previously, we reported successful posterior spine fusion using *AdBMP-7* and bone allograft^66^, and separately using *AdNull*, *AdBMP2*, *AdBMP7* and combined *AdBMP2/AdBMP7* and bone allograft^59^, with the best fusion results obtained delivering the treatment capable of generating BMP2/7 heterodimers. However, in this intradiscal delivery model, successful fusions were achieved *only* in the BMP2/7 HT group, suggesting this anterior fusion model may require a stronger osteoinductive presence for success. The BMP heterodimer osteoinduction mechanism requires further investigation.

Our most striking finding was the resistance of the IVD to BMP-induced fusion bone, and the impressive IVD/NP preservation irrespective of treatment. Although trace fusion bone may have formed in the anterior-most AF lamellae, the deeper AF and NP had no suggestion of bone formation. All significant bone produced was in the exposure zone anterior and anterolateral to the spine, consistent with prior IVD BMP studies^48–50, 52^. Despite previous reports in clinical and animal studies, bone was not induced far anterior to involve vascular or visceral organs^73^, and none laterally involving lumbar nerve roots or posteriorly to compromise the spinal canal^31–34, 52^. Several mechanisms could explain the induction of fusion bone induced in such a restricted location: (1) the damaged soft tissue in the exposure zone was where the osteoprogenitor cells that responded to the osteoinductive signals resided^84–87^, (2) the implanted BMP-expressing cells may have leaked out of the IVD and into the fusion location^43, 44^, (3) the inner portions of the IVD were too avascular to allow bone induction/formation^88–91^, or (4) apart from vascularity, the IVD acted as a barrier to neo-osteogenesis^40, 45–47^, either through production of and signaling by specific proteoglycans in the extracellular matrix or cell membrane (such as the glypicans)^92–94^, or through less-well characterized direct/active anti-osteogenesis signaling^45, 95–97^. Which of these mechanisms, or combinations thereof, best explains the fusion bone localization will require future investigation possibly delivering traceable gene-expressing cells, genes to drive neo-angiogenesis in the IVD (vascular endothelial growth factor), or enzymes to degrade the IVD matrix and/or disrupt NP signaling. The observed preservation of the NP compartment of the disc helps to explain how induced fusion bone was excluded from the IVD, but raises further questions regarding why the discs remained so nearly normal. Our endplate perforations were anticipated to result in rapid IVD degeneration and potentially fusion^98^, but neither were observed by 12 weeks. Disc preservation after LACZ gene/cell-delivery suggests that BMP-dependent disc preservation is not the mechanism. The absence of IVD degeneration and ossification may reflect the lack of bipedal spine loading, the study duration being too short, or possibly the application of the bone marrow stromal cells regardless of genetic modification. Although the mechanism for IVD preservation and/or exclusion of bone formation from the disc space was not defined in this study, we conclude that the NP/IVD blocks intervertebral bone formation and fusion within the IVD.

A major strength of our study was the use of intermittent NIAD assessment, which allowed us to measure the relative kinetics of spinal stiffening that occurred over the course of the study, without requiring euthanasia of large numbers of animals at each time point. This method limited the numbers of animals required to study spine fusion to those needed for final assessment, plus any others required for histological assessments in time course. At 12 weeks NIAD correlated significantly with IVAD and the formal biomechanics endpoints. The IVAD (q of Supplementary Fig. S2) is directly related to the bending stiffness (EI) through the equation *d*^EI^ = (1/2 DF x^2^ * y)/EI_sample_ as we have reported^71^. Based on geometry f = 1/2 q, and tan(f) = *d*^IVAD^/a, where a = ½ outer support distance. Error is introduced into interpreting *d* identically in both of these equations, as *d*^EI^ is measured after adjustment for creep, but *d*^IVAD^ does not correct for creep. However, if an adjusting scalar is added to the ƒ(f) for *d*^IVAD^, the average error equating IVAD to EI was reduced to 30% for extension. Such an error magnitude would require experimental treatment effects large enough to exceed the error. IVAD is similar to the approach successfully used by Muschik et al. (2000) in which experimentally-fused spinal segments were immobilized on one end, loaded with a specified flexion or extension load on the other end, and the induced radiographic vertebral body alignment change was measured and interpreted as an objective endpoint reflecting relative stiffness^50^. NIAD and IVAD assessments are simple to perform and offer objective measurement of relative induced spinal stiffness. NIAD can be used as a non-lethal method to follow relative spinal stiffness in small animals over the course of an experiment.

Another strength of our study is our multimodal fusion assessment. Prior descriptions of IVD fusion models^48, 50, 51, 98–101^ have non-uniformly used multiple fusion endpoint assessments. Clinically, the fusion “gold standard” has been surgical exploration and mechanical loading of the segments in question, with radiographic criteria-based methods considered more prone to false-positive or false negative errors^102^. For comparative models palpation also is the “gold standard”^103, 104^, despite the potential for subjectivity for unblinded raters. The use of objective mechanical testing, in which forces are applied and specimen deflections or stiffnesses are measured, is not commonly reported for IVD fusion studies, which can lead to false positive radiographic/histology “fusions”^50, 101^. We illustrate this point further (Fig. 4) with lateral high-definition x-rays of animals coded as “Moderate” or greater for their bone production that did not correlate with palpation or dichotomous radiographic fusion status, and mid-sagittal micro-CT cuts in those animals undergoing micro-CT that had fusions. Many of the images demonstrate considerable induced fusion bone that were palpation rated as fused in some samples, but in other samples residual motion was detectable. We employed a blinded 3-rater palpation assessment with fusion decided by majority in an effort to minimize inter-rater influence and decrease the potential for a single rater to systematically skew results by their individual rating style^105, 106^, and four-point bending tests to examine the specimens as objectively as possible.

Several limitations exist in our study. We did not track the cells that were implanted, and cannot comment on their viability over time or their location at the time of gene expression. Our ex vivo gene-therapy method also did not permit control or quantification of the level or duration of gene expression. Lack of more uniform statistical significance for the HT group was attributed to the infrequency of the very stiff 2-level fusions and subsequent effects on treatment group interquartile range and error estimates. Furthermore, achieving more double-level fusions in the HT group would have strengthened several of the correlations reported and might have enabled use of parametric statistics. We powered the study to assess fusion by palpation, and used a majority of samples for destructive biomechanical testing. Having samples for histology and micro-CT at intermediate times (4 and 8 weeks) may have elucidated the process of bone formation in our model. Additional histology and micro-CT samples at 12 weeks, including samples from all treatment groups, may have identified if anterior AF lamellae or the cartilage clefts present in some fusion masses were involved in producing the fusion bone.

What we have described is a proof of concept, and a work in progress, towards what is designed to be a percutaneous/minimally-invasive technique for human anterior spine fusions. If this exact technique was implemented today, there would be major concerns, including: requirement for a large open/maximally-invasive anterior approach, iatrogenic kyphogenesis at treated levels, and potentially different efficacy for, or leakage of treatments delivered to, the degenerated discs that are more likely to be clinical targets for such a treatment. Regarding the approach, efficient percutaneous access of two discs in a rodent for the number of times required for this study would have been extremely challenging, however, percutaneously accessing the disc in humans is well described^107–110^, and will be utilized instead of open techniques. Furthermore, as important as the sagittal plane has been shown to be in spinal surgery, a fusion technique driving undesired kyphosis would be a big problem. We suggested the kyphosis encountered in this model was caused by the differential *lack* of anterior growth, mediated by the fusion tether causing growth arrest, and *preserved* posterior growth through the disc complex (epiphysis-disc-epiphysis). This is very similar to the posterior tether and anterior overgrowth mechanism used to explain the crankshaft phenomenon in young children that have spine fusions^111^. So, it would seem that kyphogenesis would only be a concern in the population of patients that was still growing (children and adolescents), and would be avoidable if we are able to use the technique in older patients, or to further refine our model to engineer bone growth inside the disc space (fusing epiphysis to epiphysis) as originally intended. As regards use of the technique in degenerating instead of normal discs, it should be easier to get our method to work in a setting of degeneration, as the described anti-osteogenic^45–47, 112–114^ NP cells would be less populous in the degenerating tissue, weakening their interference of induced mineralization or bone formation^115–117^. We have previously reported that cells leak out of the disc injected^43^, but this leakage does not appear to result in observable generation of heterotopic bone as assessed by high-definition AP and lateral x-rays used in this study. Further study of the potential effects of treatment leakage from the implantation site is warranted.

Using intradiscal gene delivery, reproducible lumbar spine fusions were achieved with BMP2/7 heterodimers but not in any of the other treatments. These results were demonstrated not only by manual palpation of fusion and radiographic measures, but also by three objective measures of spine stiffness and histology. We also described a non-lethal method (NIAD) to monitor spine stiffness in small animal spine fusion studies in time course. Bone induced by BMP2/7 treatment was located outside of the disc space, suggesting the disc matrix and cells are either an inert physical barrier or are an active inhibitor of bone production within the IVD. Our observations support the possibility of developing a method for percutaneous human anterior spine fusion. Future investigation must address engineering osteoinduction within the IVD, assessing the feasibility of allogeneic cells for implantation, and evaluating the success of the technique in larger comparative models.

## Supplementary Information

Supplementary Information.

## Data Availability

Data generated or analyzed during this study are available from the corresponding author on reasonable request.
