# Supplementary material for: Lumbar spine intervertebral disc gene delivery of BMPs induces anterior spine fusion in lewis rats
Source: Sci Rep. 2022 Oct 7;12:16847. doi: 10.1038/s41598-022-21208-1 (PMC9547004; doi:10.1038/s41598-022-21208-1)
Supplement: Supplementary file 1 — Supplementary Information. [file 41598_2022_21208_MOESM1_ESM.docx]

**Lumbar Spine Intervertebral Disc Gene Delivery of BMPs Induces Anterior Spine Fusion in Lewis Rats**

Matthew E. Cunningham MD PhD^1,2^, Natalie H. Kelly PhD^1^, Bernard A. Rawlins MD^1,2^, Oheneba Boachie-Adjei MD^1,2^, Marjolein C. H. van der Meulen PhD^1,3^, and Chisa Hidaka MD^1^

1 HSS Research Institute, Hospital for Special Surgery, 515 E 71^st^ Street, New York, NY 10021

2 Weill Cornell Medical College, New York City, 1300 York Avenue, Lc501, New York, NY 10065

3 Meinig School of Biomedical Engineering and Sibley School of Mechanical & Aerospace Engineering, Cornell University, Ithaca, NY 14853

**a) b)**


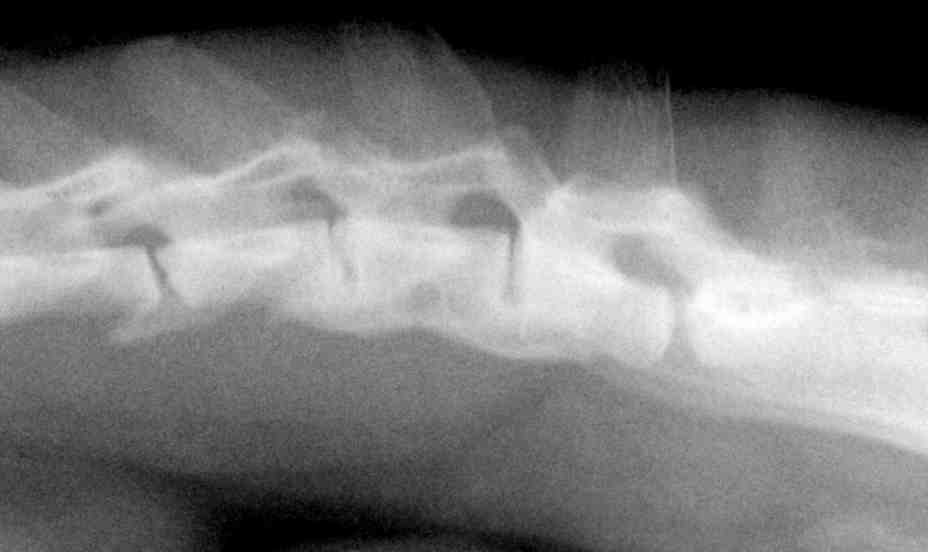

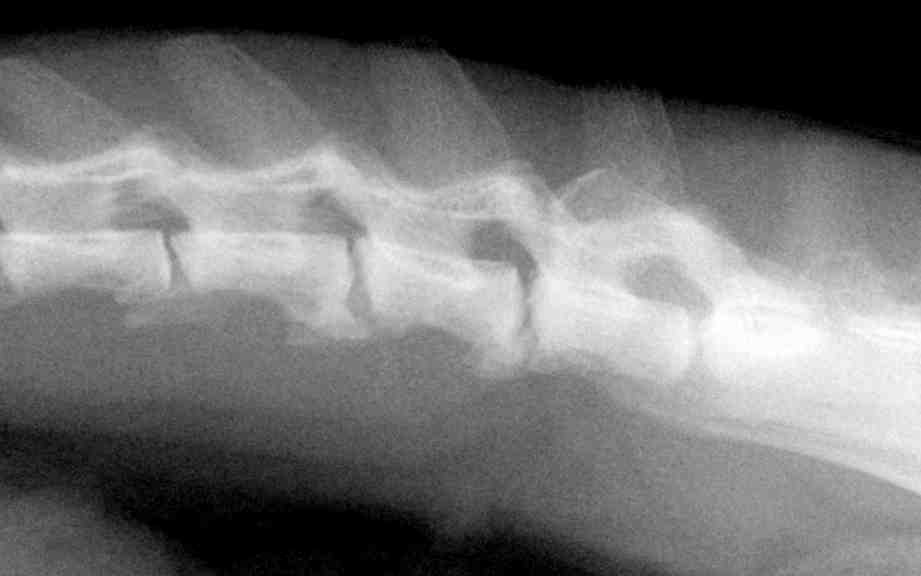

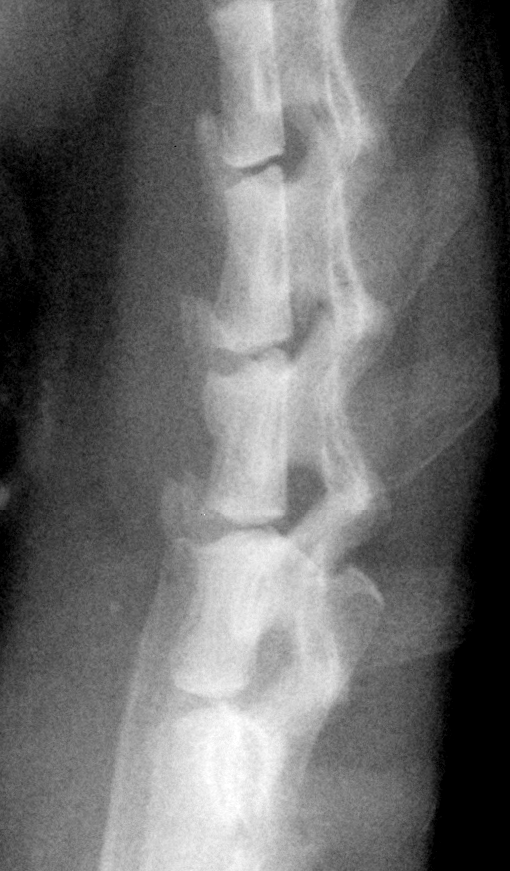
 **
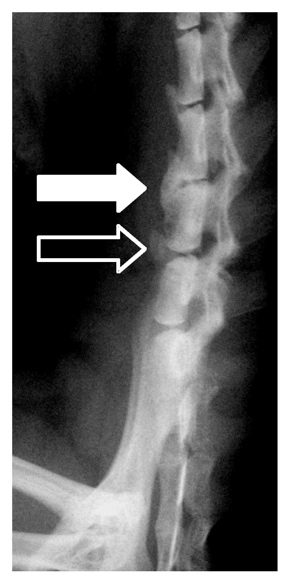
**

**0**

**1**

**2**


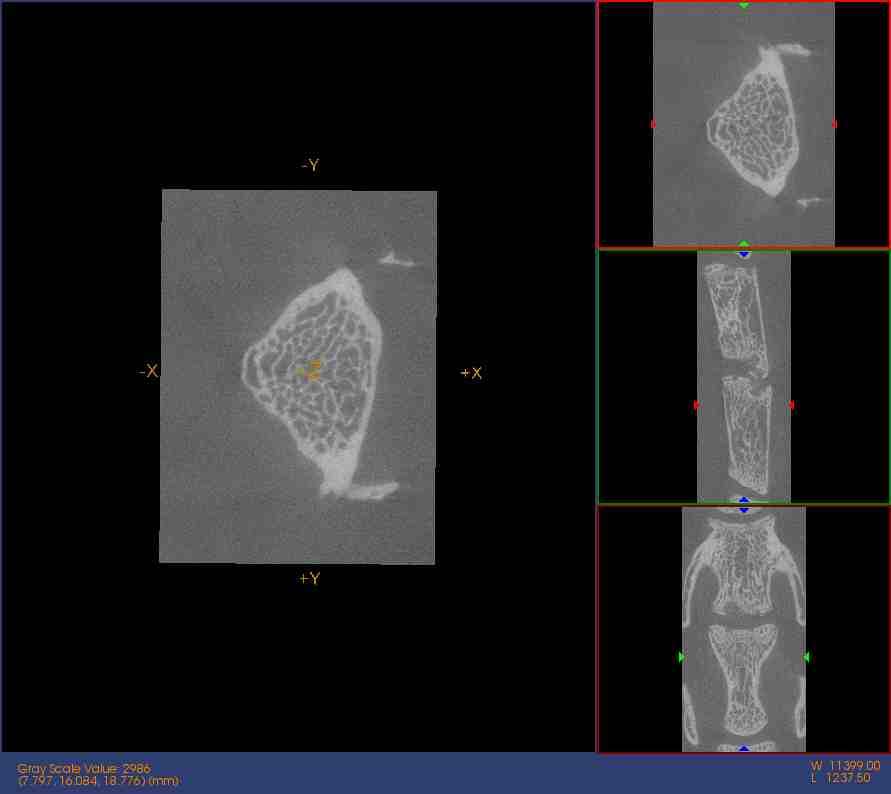


**c) d) e)**


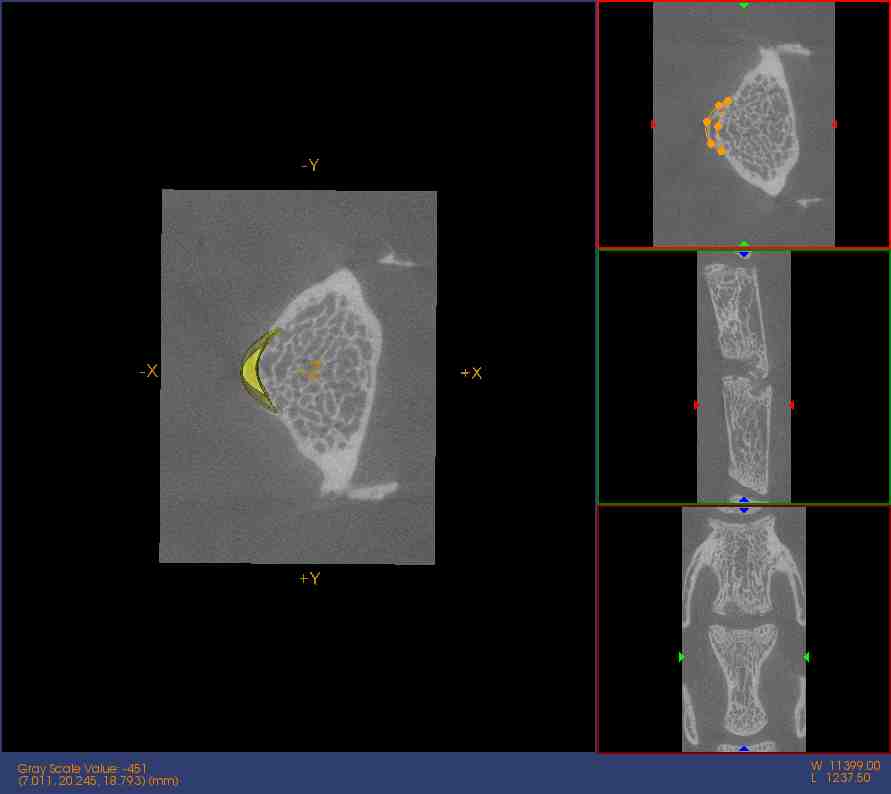

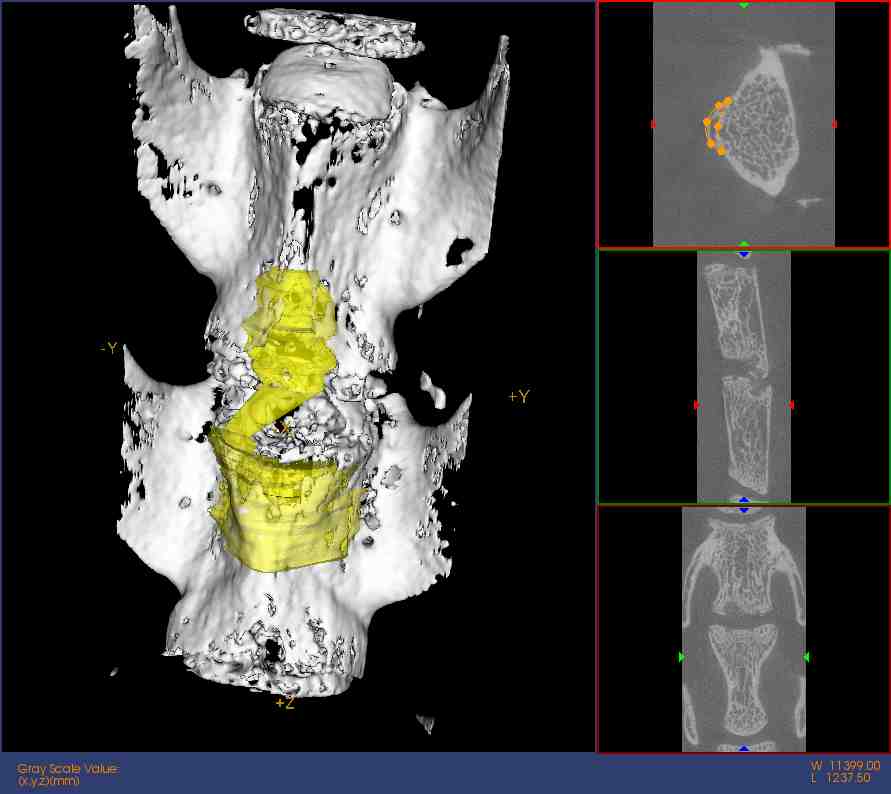


Supplementary Figure 1. Fusion and bone formation assessment using faxitron (a, b) and micro-CT (c-e). Arrows [➨] in faxitron lateral images (a, b) indicate the L4/5 (upper) and L5/6 (lower) discs. (a) Graded fusion assessment: (left image) example of a BMP2 & BMP7 homodimer-treated spine (specimen 29) graded as 0 for both levels, showing minimal bone production at both levels and no fusion at either level; (middle image) example of a BMP2-treated spine (specimen 97) graded as 1 for both levels with moderate bone production and possibility of fusion; (right image) an example of a BMP2/7 heterodimer-treated spine (specimen 45) graded as 2 for both levels with abundant bone production and probable fusion of both levels. (b) Categorical fusion assessment: example of a BMP2/7 heterodimer-treated spine (specimen 50) graded as 1 (fused) at L4/5 (upper arrow [➨]), and graded as 0 (not fused) at L5/6 (lower arrow [🢥]). Note that bone formation is external to the disc space. (c) A micro-CT axial section of a BMP2 & BMP7 homodimer-treated L4/5 vertebra showing baseline cortical bone (large arrows [▲]) and induced fusion bone (small arrows [△]). (d) An example of a 2D fusion bone region of interest with the ROI - bounded by the orange line and dots [-⏺-] drawn around the induced fusion bone in the same axial image as (c). (e) The resultant 3D ROI volume generated for the specimen after processing (yellow shaded areas) used for quantification of induced fusion bone.

**
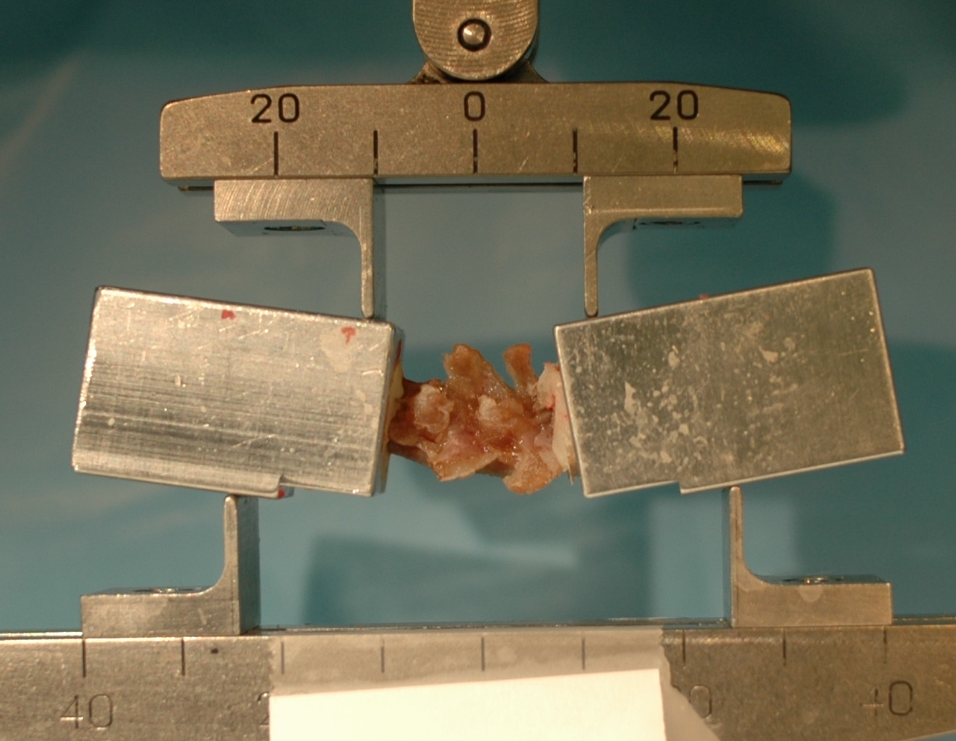
a)**

θ

d

a

φ

**b)**

**
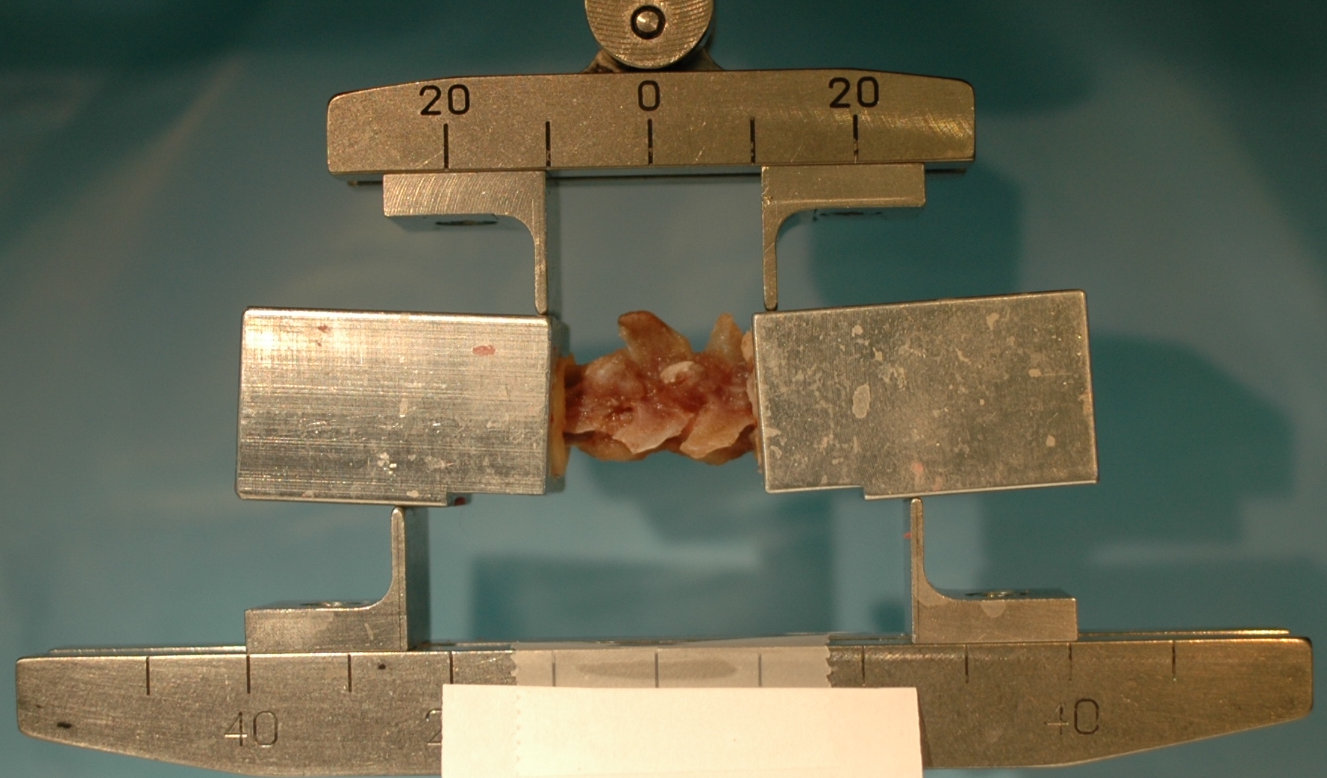
**

Supplementary Figure 2. Fusion assessment by biomechanical testing. (A) Example of LACZ (marker gene)-treated lumbar level 4-6 spinal segment tested in four-point bending in extension photographed at maximal loading/deflection; IVAD (θ), mechanical displacement (d), moment arm at zero deflection (a), and angle of deflection generated during four-point testing (φ). Four-point bending stiffness (EI) is found using the equation d = (1/2 ΔF x^2^ * y)/EIsample, (where ΔF = force applied, y = ½ inner support separation difference, x = ½ (outer support separation – inner support separation) and d = the creep re-zeroed sample displacement during the test).^65^ (B) Example of a BMP2/7 heterodimer-treated specimen with 2-level fusion at maximal loading, illustrating the low deflection observed in the stiffest specimens.
